# Trends in the disparities and equity of the distribution of traditional Chinese medicine health resources in China from 2010 to 2020

**DOI:** 10.1371/journal.pone.0275712

**Published:** 2022-10-10

**Authors:** Rixiang Xu, Tingyu Mu, Yulian Liu, Yaping Ye, Caiming Xu

**Affiliations:** 1 School of Humanities and Management, Zhejiang Chinese Medical University, Hangzhou, Zhejiang Province, China; 2 School of Nursing, Zhejiang Chinese Medical University, Hangzhou, Zhejiang Province, China; 3 Ningbo Municipal Hospital of TCM Affiliated Hospital of Zhejiang Chinese Medical University, Ningbo, China; 4 School of Law, Zhejiang University City College, Hangzhou, China; Northeastern University (Shenyang China), CHINA

## Abstract

**Background:**

At present, improving the accessibility to traditional Chinese medicine (TCM) health resources is an important component of China’s health policy. This study evaluated the trends in the disparities and equity of TCM health resource allocation from 2010 to 2020 to inform optimal future local health planning and policy.

**Method:**

The data for this study were extracted from the China Health Statistical Yearbook (2011–2021) and China Urban Statistical Yearbook (2020). The equity and rationality of the allocation of TCM health resources at the national and provincial levels were evaluated using the Gini coefficient and the health resource aggregation degree, respectively.

**Result:**

The number of TCM-related institutions, beds, health staff, outpatients and admissions increased by 1.97, 2.61, 2.35, 1.72 and 2.41 times, respectively, between 2010 and 2020. The population-based Gini coefficients for health staff, beds and institutions were 0.12, 0.23 and 0.13, respectively, indicating acceptable equity, while the geographical area-based Gini index for health staff, beds and institutions were 0.65, 0.62 and 0.62, respectively, indicating serious inequity. The agglomeration degree as a function of geographical area was as follows: eastern region > central region > western region. Moreover, the institutional and health staff gaps between the geographical areas increased from 2012 to 2020. In addition, there was a relatively balanced agglomeration degree based on the population in these three regions and an increasingly equitable allocation of institutions and health staff.

**Conclusion:**

In recent years, China’s TCM health resources and services have increased rapidly, but their proportions within the overall health system remain low. The equity and rationality of TCM health allocated by the population was better than that by the geographic area. Regional differences and inequalities, especially for institutions, still exist. A series of policies to promote the balanced development of TCM need to be implemented.

## 1. Introduction

Traditional Chinese Medicine (TCM) is an indispensable part of traditional Chinese culture. After 2,500 years of exploration and practice, TCM is generally considered to have significant application value in the life sciences [1]. TCM is characterised by four ways of diagnosing diseases (look, listen, question and feel the pulse), several main treatment methods, including Chinese herbs, massage, acupuncture, cupping and guasha, and the vital preventive medicine approach to “treating diseases before they arise”. There are significant practical and theoretical differences in the diagnosis and treatment of diseases with TCM as compared to Western medicine [2]. Although some still question the validity of TCM, TCM has been proven effective in the treatment of chronic diseases and, more recently, COVID-19 [3–6]. At present, due to its unique advantages, TCM is widely used in 183 countries [7]. In Japan, South Korea, Europe and the United States, TCM is now an important component of the health system [8].

In China, the development of TCM experienced many obstacles after China’s Economic Reform and Open Up (a TCM modernization movement) [9]. Firstly, because of its clear mechanism of treatment and remarkable effect in many diseases, Western medicine quickly became mainstream medicine. As a result, the clinical space occupied by TCM was severely reduced [10]. Secondly, with the rapid development of society, a large number of new diseases and phenomena that are difficult to explain by TCM theory were observed in clinical practice [9]. As such, a group of scholars and authoritative experts questioned the operational logic and effectiveness of TCM from the perspective of modern science [11]. In this context, and with the current rapid growth of China’s healthcare system, not only is TCM development stagnant but TCM faces the risk of being replaced by Western medicine. Together, these factors have led to low levels of medical resource utilization, irrational health resource allocation and low operational efficiency of TCM institutions.

In 2009, healthcare reform was initiated by the Chinese government. As part of this reform, a series of policies were developed to enforce adherence to the principle that TCM and Western medicine are equally important in the health system, support the development of TCM, and strengthen the construction of, and financial investment in, TCM hospitals [7]. In 2012, the National TCM Bureau launched the “grass-roots TCM service capacity improvement project”, which aimed to improve the coverage of TCM health services and the support of medical insurance for patients to utilise TCM health services. This policy had important implications for improving the availability and affordability of TCM health services [12]. In 2015, the National TCM Bureau implemented the”reform of TCM hospitals” project across the country with the aim of improving the allocation of TCM health resources, standardizing the construction of county-level hospitals, and exploring a medical insurance payment model that matched the characteristics of TCM, in order to further increase the importance of TCM in the health system [13]. Although the development of TCM has made remarkable gains under this series of positive policies, there remain problems such as insufficient TCM resources and shrinking TCM services. In 2016, the "TCM Development Plan (2016–2030)" was implemented to clarify the specific goals for the development of TCM health resources, such as the provision of TCM services to everyone by 2020 and the full coverage of TCM services by 2030 [14]. This is the first long-term strategic plan for the development of TCM and provides clarity regarding the direction of TCM over the next 15 years.

However, the effectiveness of these policies requires verification through further research. Although previous studies have focused on the allocation of TCM health resources, they have not covered the key time node of the policy (five-year plan) [15]. Thus, this study collected the latest official data and calculated the Gini coefficient and agglomeration degree to analyse the trends in equity at the national and regional levels from two dimensions: population and geography. Moreover, this research covers two five-year plans for the development of TCM (2011 to 2015 and 2016 to 2020), and thus, provides a more accurate reflection of the effectiveness of these policies.

## 2. Methods

### 2.1 Data sources

The China Health (Family Planning) Statistical Yearbook is a comprehensive national-level health service statistics manual edited by the National Health Commission. This study collected and analysed data from the China Health Statistical Yearbook between the years 2011 and 2020. It should be noted that the data in each edition of the yearbook is from the previous year. TCM institutions (TCM hospitals, integrated Chinese and Western hospitals, ethnic medicine hospitals, TCM clinics and TCM research institutions), TCM health staff (practising TCM doctors, assistant TCM practising doctors, trainee TCM practitioners and TCM pharmacists), bed numbers (beds in TCM institutions and clinical departments of TCM in other health institutions) and the number of outpatient visits and admissions (the available statistics include TCM institutions and clinical departments of TCM in other health institutions) were obtained as indicators. These data covered 31 provinces in mainland China, excluding Hong Kong, Macau and Taiwan, due to the inconsistent statistical standards. Due to changes in the statistical calibre of the 2012 edition of the yearbook, the indicator data (mentioned above) in 2011 and 2010 at the provincial level are missing.

### 2.2 Data processing

Two data analysts independently extracted and entered the data into a pre-programmed Excel Workbook, including the above-mentioned indicators at the national and provincial levels each year from 2010 to 2019. The per capita level for each indicator was calculated by dividing the total for each indicator by the local population for the year. The aim of this was to reflect the availability of TCM health resources at an average level.

### 2.3 Statistical analysis

Descriptive statistical methods were used to analyse the proportion of TCM input resources (institutions, beds and health staff) and output services (outpatient visits and admissions) relative to the total health resources and health services.

Lorenz curves and the Gini coefficients (G) were used to determine the equality of TCM health resource allocation. Lorenz curves provide visualisation of the degree of equality of resource allocation. Taking the indicator of health staff as an example, the x-axis on the Lorenz curve is the cumulative population (geographical area) proportion of the 31 provinces (sorted by the number of beds per capita from small to large) and the y-axis is the cumulative proportion of beds in the corresponding province. The closer the curve is to the x-axis, the more inequality there is. The line corresponding to the function y = x is regarded as an absolute equality curve. G is calculated as the area between the Lorenz curve and the perfect equality curve divided by 1/2. The size of the value directly reflects the degree of equality of resource allocation; the closer it is to 1, the more inequality. The formula used to calculate G is as follows:

$$G=\sum_{i=1}^{n}{W_{i}Y}_{i}+2\sum_{i=1}^{n}{(W}_{i}-W_{i}V_{i})‐1$$ (1)

where W_i_ and Y_i_ are the proportions of the population (geographical area) and TCM health resources of each province to the total population (geographical area) and total TCM health resources, respectively; V_i_ represents the cumulative proportion of each indicator in the 31 provinces (sorted by the number of each indicator per capita from small to large). G < 0.2 is regarded as absolute equality, 0.2 < G < 0.3 as relative equality, 0.3 < G < 0.4 as proper equality, 0.4 < G < 0.5 as relative inequality and G > 0.5 as serious inequality *[15]*. Stata 26.0 and EXCEL 2016 were used to analyse these data.

The health resources agglomeration degree (HRAD) was used to evaluate the rationality and equity of the allocation of health resources in different provinces [16]. The formula below was used to calculate the geographic area-based and population-based HRADs of TCM health resources.

HRADAi=HRiHRnAiAnorHRADPi=HRiHRnPiPn (2)

where HR_i_ is the health resource quantity of the ith region; A_i_ is the geography of the ith region; P_i_ is the population of the ith region. HRAD_Ai_ and HRAD_Pi_ refer to HRAD based on the geographic area and HRAD based on the population of the ith region, respectively. The closer HRAD is to 1, the more equitable the allocation of health resources is in this area.

## 3. Results

### 3.1 Trends in the development of TCM health resources and services

Table 1 shows the changes in TCM health resources and services over the 11 years following the implementation of healthcare reform. At the national level, China’s health institutions, beds and health staff increased by 1.09 times, 1.904 times and 1.63 times, respectively. Among these, institutions, beds and health staff related to TCM increased by 1.97, 2.61 and 2.35 times, respectively. Moreover, the number of outpatient visits and hospital admissions as health output indicators increased by 1.51 times and 1.93 times, respectively; the number of TCM outpatient visits and TCM hospital admissions increased by 1.72 and 2.41 times, respectively. The proportions of the input and output indicators related to TCM relative to the total indicators continuously increased during the period, with institutions increasing from 3.92% to 7.07%, beds from 11.46% to 15.74%, health staff from 12.17% to 17.49%, number of inpatient visits from 14.65% to 16.75%, and number of admissions from 15.20% to 19.09%.

**Table 1:** Trends of TCM health resources and services from 2010–2020.

Year	Health staffs (thousands)		Beds (thousands)		Institutions (thousands)		Number of outpatient visits (millions)		Number of admissions (millions)	
	Total	TCM(%)	Total	TCM(%)	Total	TCM(%)	Total	TCM(%)	Total	TCM(%)
2010	2899.9	353.0(12.2)	4786.8	548.7(11.5)	936.9	36.7(3.9)	4180.6	612.6(14.7)	95.2	14.5(15.2)
2011	3007.4	378.0(12.6)	5159.9	618.2(12.0)	954.4	38.2(4.0)	4479.2	675.3(15.1)	107.5	16.7(15.5)
2012	3180.2	488.4(15.4)	5724.8	705.8(12.3)	950.3	39.4(4.1)	4961.3	747.0(15.1)	127.3	20.2(15.9)
2013	3393.3	522.5(15.4)	6181.9	794.2(12.9)	974.4	42.0(4.3)	5301.8	814.1(15.4)	140.1	22.8(16.3)
2014	3522.9	545.3(15.5)	6601.2	877.3(13.3)	981.4	43.6(4.5)	5615.6	874.3(15.6)	153.8	25.4(16.5)
2015	3687.7	580.4(15.7)	7015.2	957.5(13.7)	983.5	46.5(4.7)	5799.4	909.1(15.7)	160.9	26.9(16.7)
2016	3851.2	612.7(15.9)	7410.5	1033.5(14.0)	983.4	49.5(5.0)	6079.1	962.3(15.8)	175.3	29.5(16.8)
2017	4053.2	663.6(16.4)	7940.3	1135.6(14.3)	986.6	54.2(5.5)	6393.8	1018.9(15.9)	189.2	32.9(17.4)
2018	4279.6	714.9(16.7)	8404.1	1234.2(14.7)	997.4	60.7(6.1)	6635.9	1071.5(16.2)	200.2	35.8(17.9)
2019	4543.2	767.2(16.9)	8807.0	1328.8(15.1)	1007.6	65.8(6.5)	7115.3	1163.9(16.4)	211.8	38.6(18.2)
2020	4738.1	828.9(17.5)	9100.7	1432.9(15.7)	1022.9	72.4(7.1)	6313.5	1057.6(16.8)	183.5	35.0(19.1)

**S1 Table** presents the number of TCM health resources for each province in 2020 and their increments from 2012 to 2020. In terms of geographic distribution, Inner Mongolia, Gansu (the lowest GDP per capita) and Beijing (the highest GDP per capita), respectively, ranked highest in TCM institutions, beds and health staff per capita in 2019. Shandong, Chongqing and Beijing, respectively, had the highest increases in TCM institutions, beds and health staff per capita from 2012 to 2019. On the contrary, Shanghai (2^nd^ GDP per capita) ranked lowest in terms of the number of TCM institutions and beds per capita, while Anhui had the lowest health staff per capita in 2019. From 2012 to 2019, the lowest increases in TCM institutions, beds and health staff per capita were in Gansu, Shanghai and Jiangxi, respectively.

### 3.2 Equity in the allocation of TCM health resources

**Fig 1:**
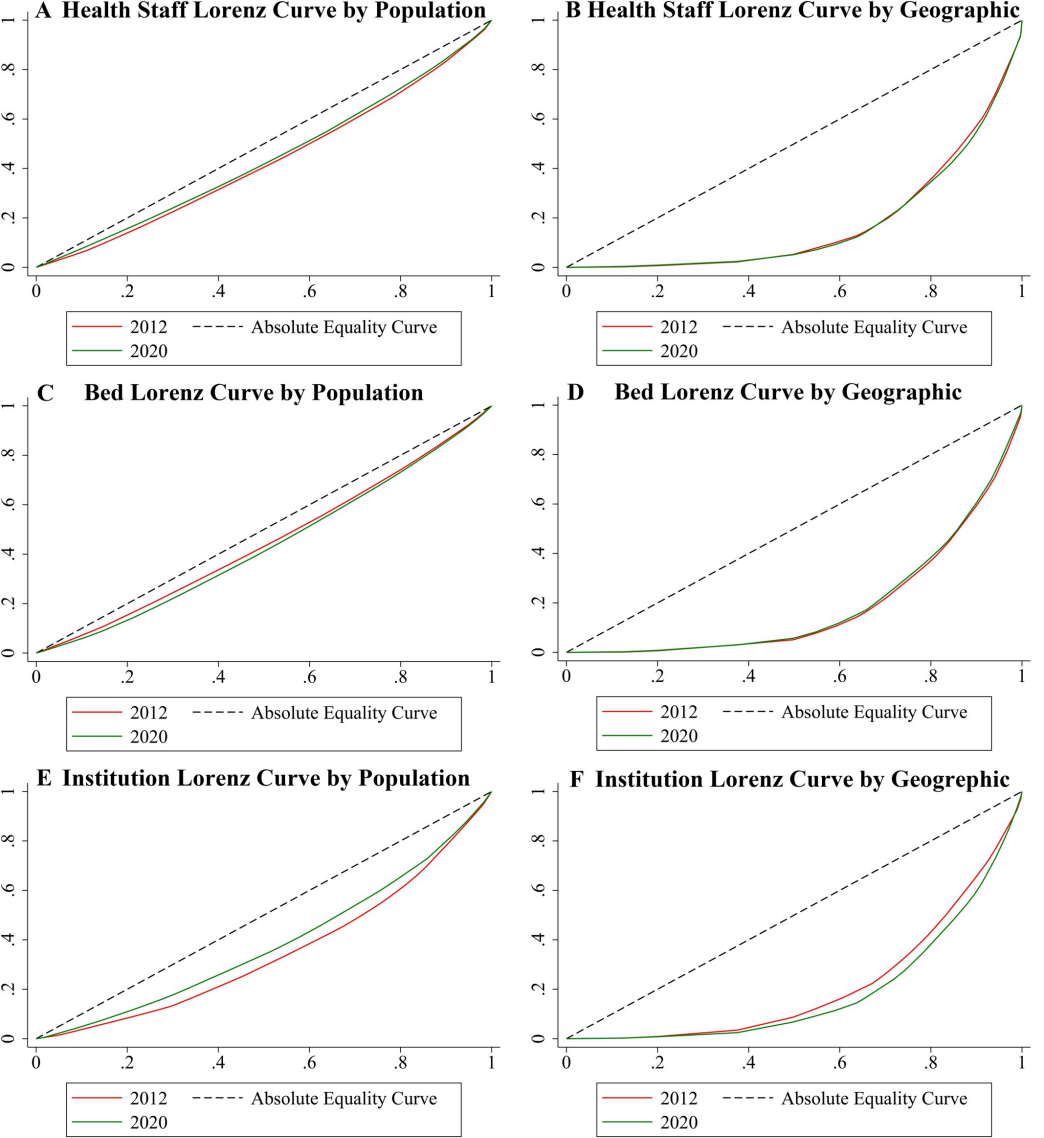
The Lorenz curves and Gini coefficients changes of the three indicators.

**Table 2** shows the G index of the three indicators against population size and geographical area in different years. From a population perspective, the G index of institutions reduced from 0.302 to 0.232, representing an increase from proper equality to relative equality. The G index of beds increased from 0.101 to 0.130, indicating that although equity was reduced, it was still within the absolute equity range. The G index of health staff decreased from 0.145 to 0.119, representing increasing equity. However, the G indices as a function of geographical area were all greater than 0.6 in 2020, which indicates poorer equity. **Fig 1** presents the Lorenz curves and the changes in the G index of the three indicators. The institution and health worker curves based on population are closer to the absolute equality curve, whereas the bed curve is farther away from the absolute equality curve. In contrast, the three indicator curves based on geographic area show the opposite performance.

**Table 2:** The Gini coefficients of TCM health resources from 2012 to 2020.

Years	Allocation by population			Allocation by geographic		
	Staff	Institution	Bed	Staff	Institution	Bed
2012	0.145	0.302	0.101	0.645	0.573	0.629
2013	0.141	0.285	0.102	0.648	0.581	0.625
2014	0.138	0.279	0.108	0.649	0.584	0.622
2015	0.140	0.280	0.109	0.649	0.589	0.622
2016	0.136	0.276	0.113	0.649	0.593	0.622
2017	0.133	0.265	0.116	0.651	0.599	0.620
2018	0.133	0.260	0.121	0.651	0.608	0.619
2019	0.130	0.246	0.126	0.651	0.618	0.616
2020	0.119	0.232	0.130	0.653	0.620	0.617

**Tables 3 and 4** show the HRADs of the three input indicators based on geographic area and population, respectively, in Eastern, Central and Western China. From the perspective of geographic area, the HRADs of institutions, beds and health personnel in the Eastern region were all greater than 3, while those in the Western region were all lower than 0.5, indicating that TCM health resources were concentrated in the Eastern region. Moreover, there was a trend toward greater concentration of institutions and health staff in the Eastern region between 2012 and 2020. From the population perspective, the HRADs between the three regions were very close to 1, indicating reasonable allocation of TCM health resources. In addition, the gap in the HRADs of institutions and health staff between the three regions shrunk. S2 Table presents the geographic area-based and population-based HRADs for each province in 2020. The results clearly shows that the closer to the Eastern region (except Chongqing), the higher the HRAD based on geographic area. However, population-based HRADs across provinces were random and balanced.

**Table 3:** The geographic area-based agglomeration degree of TCM health resource from 2012–2020.

Years	Institution			Bed			Health staff		
	Eastern	Central	Western	Eastern	Central	Western	Eastern	Central	Western
2012	2.89	1.55	0.57	3.47	1.77	0.42	3.76	1.63	0.41
2013	2.99	1.50	0.56	3.4	1.76	0.44	3.78	1.61	0.41
2014	3.07	1.49	0.55	3.35	1.76	0.45	3.79	1.61	0.41
2015	3.12	1.48	0.55	3.33	1.77	0.45	3.81	1.58	0.42
2016	3.19	1.45	0.55	3.3	1.77	0.45	3.83	1.57	0.42
2017	3.32	1.43	0.53	3.29	1.75	0.46	3.88	1.54	0.41
2018	3.48	1.39	0.51	3.23	1.76	0.46	3.91	1.53	0.41
2019	3.56	1.37	0.51	3.18	1.75	0.47	3.93	1.5	0.42
2020	3.54	1.45	0.49	3.13	1.79	0.47	3.89	1.54	0.41

**Table 4:** The population-based agglomeration degree of TCM health resource from 2012–2020.

Years	Institution			Bed			Health staff		
	Eastern	Central	Western	Eastern	Central	Western	Eastern	Central	Western
2012	0.78	0.86	1.50	0.94	0.98	1.12	1.02	0.90	1.09
2013	0.81	0.84	1.49	0.92	0.98	1.15	1.02	0.90	1.09
2014	0.83	0.83	1.46	0.90	0.98	1.17	1.02	0.89	1.09
2015	0.84	0.83	1.44	0.90	0.99	1.17	1.03	0.88	1.09
2016	0.86	0.81	1.44	0.89	0.99	1.19	1.03	0.88	1.10
2017	0.89	0.8	1.39	0.88	0.98	1.20	1.04	0.87	1.09
2018	0.94	0.78	1.35	0.87	0.99	1.21	1.05	0.86	1.09
2019	0.96	0.77	1.33	0.85	0.99	1.24	1.05	0.84	1.10
2020	0.92	0.85	1.29	0.81	1.05	1.24	1.01	0.91	1.09

## 4. Discussion

This study used publicly available data from the National Health Statistical Yearbook to comprehensively analyse the development trend in TCM over the 10 years following the implementation of healthcare reform in China. Several key findings were obtained. First, the growth rate of TCM health resources and services was higher than the overall growth rate. Second, the proportion of TCM health resources and services at the overall level steadily increased during the period. Third, the three input indicators (institutions, beds and health staff) developed at different speeds in different provinces, and their development did not seem to correlate with the level of the economy. Fourth, the equity of the allocation of TCM institutions and health staff in different provinces gradually improved, while that of TCM beds showed the opposite trend.

At the beginning of the 19th century, many Western academic ideas were introduced into China, a process known as the Eastward transmission of Western sciences [17]. Due to the development and remarkable effects of modern Western medicine and the clear underlying mechanisms, it was rapidly accepted by Chinese people and became mainstream medicine. This, together with the release of a series of biased policies, meant that TCM was facing a catastrophic existential crisis. Fortunately, in recent decades, China has rediscovered the irreplaceable value of TCM in the health system. Following healthcare reform, a series of positive policies were issued to promote the development of TCM in China [7]. The proportion of the health system and services occupied by TCM is increasing, and the numbers of TCM-related beds, institutions, health staff, outpatient visits and admissions have increased by 1.73 to 2.41 times. However, the number of TCM health resources and services was still less than a quarter of that of Western medicine in 2020. Over time, the marginal effect of TCM development will decrease. According to a comparison of the annual growth of TCM health resources relative to that of Western medicine, it is predicted that TCM-related beds and health staff will reach a peak of 25–30% of that of Western medicine. At present, there are still many barriers to the development of TCM, including the unreasonable structure of the Chinese government’s financial investment. A previous study reported that the financial allocation to TCM institutions accounted for only 2.90% of that of all health institutions in 2018 [18]; this is a serious mismatch given that the number of TCM institutions accounts for 6.09% of all health institutions. In addition, changes in the populations of different provinces affect the allocation of TCM health resources. For example, the rapid population loss in Northeast China (the population has decreased by more than 10 million over the past 10 years) has directly increased the growth rate per capita of TCM resources in this area, while the trend in Guangdong is the opposite [19]. Therefore, the design of future health policies also needs to take this internal population migration into account. As China’s inter-regional and intra-regional development imbalances gradually intensify, it is an inevitable trend to lead to unequal access to public resources by the public [20, 21]. However, TCM health resources seem to be an exception. The results of this study show that the population-based HRADs in regions with high economic development levels such as Shanghai and Jiangsu are relatively low. This phenomenon may indicate that TCM is not regarded as an essential public resource in some provinces or regions. Therefore, it is urgent to improve the recognition of the value of TCM health resources by governments at all levels.

China’s population and geographical distribution are unique, with the Eastern region occupying a small geographical area but containing a large population (11% of the area accommodates 43% of the population), while the Western region is characterised by a large geographical area and a small population (71% of the area accommodates 27% of the population). Therefore, if governments design health policies based on the population, the distribution of resources based on geographic area will suffer. The results of this study indicate that the current policies issued by the Chinese government aim mainly to meet the TCM health resource needs of the population, not the needs of the region. Ignoring the differences in geographic areas may lead to inequities in spatial accessibility. Nonetheless, the Chinese government is attempting to improve the spatial accessibility of public resources by building a convenient transportation network and optimizing living locations [22]. Therefore, the focus of our discussion below is mainly based on the population perspective. The number of TCM institutions per capita could best reflect the availability of local TCM health services because both health worker and bed resources must be attached to institutions. In our study, Shandong province was found to have the highest growth in TCM institutions, likely because it had a lower level of TCM institutions per capita in 2012. This phenomenon might explain why the equity of TCM institutions increased during the study period. The results also confirmed that recent Chinese government policies have played a positive role in promoting the TCM health resource balance [15]. However, the G index of TCM institutions was 0.23 in 2020, which is within the relative equity range. The main reason for this is that the allocation levels of TCM institutions in Hubei, Hunan, Anhui, Jiangxi and Jiangsu are relatively low. Therefore, the Chinese government should adopt a comprehensive strategy to increase the quantity of TCM institutions in these provinces which currently have poor accessibility to TCM institutions.

The current results also suggested that the allocation of TCM health staff in 2020 was within the absolute equity range, and that the equity was still increasing. The main contributor to this positive trend is the establishment of strong training programs for TCM talents in each province. However, previous studies have reported lower TCM health worker wages in provinces with lower economic levels, which may be a trigger for future inequities [11, 23]. Empirical evidence indicates that the primary cause of talent migration is the economy [24]. This basic market economy logic will aggravate the inequity of human resources between urban and rural areas and between different provinces. These problems must be considered when undertaking salary reform in public TCM hospitals.

Beds in TCM hospitals are indispensable resources for inpatients. Nationwide, the number of beds related to TCM increased 2.42 times, exceeding the changes in other indicators. However, the current analysis indicated that although TCM bed resource allocation was within the absolute equity range, it exhibited a decreasing trend. In particular, the HRADs of beds in Eastern provinces such as Fujian, Guangdong and Hainan were lower than in other regions in 2020. A possible explanation for this is that the high quality and quantity of Western medicine resources in the Eastern region attracts more patients who need hospitalization [25] because Western medicine has advantages over TCM in terms of hospitalization (based on the results of an interview with the director of a TCM hospital) [26]. Therefore, lower admission demands in these regions may lead to lower TCM beds per capita.

There are several limitations of this study that should be noted. First, data on TCM health resources by province in 2010 and 2011 were not available, which limited our ability to observe complete trends after the implementation of healthcare reform. Second, the measurement of equity in the allocation of TCM health resources between urban and rural areas is also an indicator of concern to society. However, analysis of this indicator could not be carried out due to the difficulty of accessing the relevant data. Third, there are several algorithms for calculating G, and the algorithm used in this study will produce slightly lower values than the actual value; this means that this study may have slightly overestimated the true equity. Fourth, this study used household registration population data, which leads to the overestimation of per capita health resources in economically developed regions and underestimation in economically underdeveloped regions. Future application of refined demographic data may improve the accuracy of the results [27].

## 5. Conclusion

This study comprehensively analysed the trends in the development of TCM over the 11 years following healthcare reform in China. From 2010 to 2020, the number of TCM-related health resources and services has roughly doubled, but their proportion relative to the entire health system was only 7.1% to 19.1%. Population-based G and HRADs were better than the geographic area-based estimates. The distribution of TCM-related institutions was at a relative equality level, with a G of 0.23. The equity of TCM-related beds showed a deteriorating trend, with the overall G increasing by 0.03 and the HARD in the eastern region decreasing by 0.13 during this period. In the future, authorities should optimise the allocation of health resources by focusing on areas with poor health resource allocation, striving to narrow the gap in TCM resources in different regions. In addition, governments at all levels should increase the financial investment and policy incentives for TCM hospitals and should formulate operating mechanisms suitable for the development of TCM.

## Supporting information

S1 Table The level and incrementation of TCM health resources per 1000 person in different provinces. (DOCX)

S2 Table The agglomeration degree of TCM health resources in different province in 2020. (DOCX)
