# Supplementary material for: Trends in the disparities and equity of the distribution of traditional Chinese medicine health resources in China from 2010 to 2020
Source: PLoS One. 2022 Oct 10;17(10):e0275712. doi: 10.1371/journal.pone.0275712 (PMC9550081; doi:10.1371/journal.pone.0275712)
Supplement: S1 Table — (DOCX) [file pone.0275712.s001.docx]

**Supplementary Table 1. The level and incrementation of TCM health resources per 1000 person in different provinces.**

| Province | Location | Institution | | |  | Bed | | |  | Health staff | | |
| --- | --- | --- | --- | --- | --- | --- | --- | --- | --- | --- | --- | --- |
|  |  | 2020 | 2012 | Increment |  | 2020 | 2012 | Increment |  | 2020 | 2012 | Increment |
| Anhui | Central | 0.034 | 0.008 | 0.026 |  | 1.009 | 0.398 | 0.611 |  | 0.502 | 0.219 | 0.284 |
| Beijing | Eastern | 0.048 | 0.039 | 0.009 |  | 1.24 | 0.827 | 0.413 |  | 1.278 | 0.874 | 0.404 |
| Fujian | Eastern | 0.048 | 0.032 | 0.016 |  | 0.697 | 0.483 | 0.214 |  | 0.561 | 0.389 | 0.172 |
| Gansu | Western | 0.066 | 0.06 | 0.006 |  | 1.776 | 0.779 | 0.997 |  | 0.74 | 0.485 | 0.255 |
| Guangdong | Eastern | 0.042 | 0.023 | 0.019 |  | 0.606 | 0.373 | 0.233 |  | 0.475 | 0.355 | 0.12 |
| Guangxi | Western | 0.043 | 0.028 | 0.015 |  | 1.037 | 0.485 | 0.553 |  | 0.52 | 0.281 | 0.239 |
| Guizhou | Western | 0.037 | 0.016 | 0.022 |  | 1.165 | 0.461 | 0.704 |  | 0.485 | 0.232 | 0.253 |
| Hainan | Eastern | 0.042 | 0.026 | 0.016 |  | 0.724 | 0.389 | 0.336 |  | 0.387 | 0.225 | 0.162 |
| Hebei | Eastern | 0.065 | 0.026 | 0.039 |  | 0.968 | 0.465 | 0.502 |  | 0.63 | 0.328 | 0.302 |
| Henan | Central | 0.027 | 0.014 | 0.013 |  | 1.094 | 0.519 | 0.575 |  | 0.547 | 0.315 | 0.232 |
| Heilongjiang | Central | 0.056 | 0.035 | 0.021 |  | 1.134 | 0.484 | 0.651 |  | 0.508 | 0.322 | 0.186 |
| Hubei | Central | 0.029 | 0.022 | 0.008 |  | 1.072 | 0.569 | 0.503 |  | 0.452 | 0.329 | 0.123 |
| Hunan | Central | 0.042 | 0.027 | 0.015 |  | 1.232 | 0.627 | 0.606 |  | 0.563 | 0.377 | 0.186 |
| Jilin | Central | 0.106 | 0.050 | 0.056 |  | 1.046 | 0.523 | 0.523 |  | 0.672 | 0.374 | 0.298 |
| Jiangsu | Eastern | 0.032 | 0.014 | 0.017 |  | 0.814 | 0.487 | 0.327 |  | 0.505 | 0.295 | 0.211 |
| Jiangxi | Central | 0.034 | 0.023 | 0.01 |  | 0.934 | 0.467 | 0.466 |  | 0.453 | 0.305 | 0.149 |
| Liaoning | Eastern | 0.062 | 0.041 | 0.022 |  | 0.951 | 0.498 | 0.453 |  | 0.53 | 0.359 | 0.171 |
| Inner Mongolia | Western | 0.129 | 0.083 | 0.047 |  | 1.592 | 0.573 | 1.019 |  | 0.933 | 0.592 | 0.341 |
| Ningxia | Western | 0.053 | 0.038 | 0.016 |  | 1.035 | 0.662 | 0.374 |  | 0.572 | 0.37 | 0.202 |
| Qinghai | Western | 0.065 | 0.045 | 0.02 |  | 1.38 | 0.662 | 0.718 |  | 0.755 | 0.416 | 0.34 |
| Shandong | Eastern | 0.055 | 0.014 | 0.042 |  | 0.968 | 0.551 | 0.417 |  | 0.63 | 0.327 | 0.302 |
| Shanxi | Central | 0.094 | 0.059 | 0.035 |  | 0.917 | 0.493 | 0.424 |  | 0.639 | 0.447 | 0.192 |
| Shaanxi | Western | 0.056 | 0.034 | 0.023 |  | 1.108 | 0.611 | 0.497 |  | 0.569 | 0.379 | 0.19 |
| Shanghai | Eastern | 0.016 | 0.011 | 0.004 |  | 0.543 | 0.396 | 0.147 |  | 0.498 | 0.329 | 0.169 |
| Sichuan | Western | 0.087 | 0.057 | 0.030 |  | 1.313 | 0.663 | 0.65 |  | 0.807 | 0.545 | 0.262 |
| Tianjin | Eastern | 0.029 | 0.014 | 0.015 |  | 0.779 | 0.505 | 0.274 |  | 0.95 | 0.504 | 0.445 |
| Tibet | Western | 0.057 | 0.033 | 0.024 |  | 0.978 | 0.314 | 0.664 |  | 0.846 | 0.388 | 0.458 |
| Xinjiang | Western | 0.046 | 0.045 | 0.001 |  | 1.221 | 0.759 | 0.462 |  | 0.475 | 0.325 | 0.15 |
| Yunnan | Western | 0.041 | 0.026 | 0.015 |  | 1.078 | 0.495 | 0.584 |  | 0.459 | 0.222 | 0.237 |
| Zhejiang | Eastern | 0.052 | 0.027 | 0.025 |  | 0.889 | 0.568 | 0.321 |  | 0.671 | 0.432 | 0.239 |
| Chongqing | Western | 0.105 | 0.062 | 0.043 |  | 1.465 | 0.501 | 0.965 |  | 0.717 | 0.451 | 0.266 |
