# Supplementary material for: Trends in the disparities and equity of the distribution of traditional Chinese medicine health resources in China from 2010 to 2020
Source: PLoS One. 2022 Oct 10;17(10):e0275712. doi: 10.1371/journal.pone.0275712 (PMC9550081; doi:10.1371/journal.pone.0275712)
Supplement: S2 Table — (DOCX) [file pone.0275712.s002.docx]

**Supplementary Table 2. The agglomeration degree of TCM health resources in different province in 2020.**

| Province | Location | Based on geographic area | | |  | Based on population | | |
| --- | --- | --- | --- | --- | --- | --- | --- | --- |
|  |  | Institution | Bed | Health staff |  | Institution | Bed | Health staff |
| Anhui | Central | 1.997 | 2.956 | 2.547 |  | 0.67 | 0.992 | 0.855 |
| Beijing | Eastern | 8.57 | 11.131 | 19.848 |  | 0.939 | 1.220 | 2.175 |
| Fujian | Eastern | 5.434 | 3.835 | 3.244 |  | 2.043 | 1.442 | 1.220 |
| Gansu | Western | 2.157 | 1.57 | 2.189 |  | 0.942 | 0.685 | 0.956 |
| Guangdong | Eastern | 0.517 | 0.702 | 0.506 |  | 1.286 | 1.747 | 1.26 |
| Guangxi | Western | 3.907 | 2.856 | 3.866 |  | 0.816 | 0.597 | 0.808 |
| Guizhou | Western | 1.199 | 1.472 | 1.277 |  | 0.831 | 1.021 | 0.885 |
| Hainan | Eastern | 1.09 | 1.717 | 1.237 |  | 0.728 | 1.146 | 0.826 |
| Hebei | Eastern | 1.678 | 1.449 | 1.338 |  | 0.825 | 0.713 | 0.658 |
| Henan | Central | 3.401 | 2.572 | 2.899 |  | 1.259 | 0.952 | 1.073 |
| Heilongjiang | Central | 2.142 | 4.378 | 3.79 |  | 0.526 | 1.076 | 0.931 |
| Hubei | Central | 0.498 | 0.514 | 0.398 |  | 1.082 | 1.116 | 0.864 |
| Hunan | Central | 1.212 | 2.241 | 1.635 |  | 0.571 | 1.055 | 0.770 |
| Jilin | Central | 1.773 | 2.601 | 2.056 |  | 0.826 | 1.213 | 0.959 |
| Jiangsu | Eastern | 1.814 | 0.903 | 1.004 |  | 2.066 | 1.029 | 1.144 |
| Jiangxi | Central | 3.342 | 4.329 | 4.651 |  | 0.618 | 0.801 | 0.86 |
| Liaoning | Eastern | 1.21 | 1.699 | 1.428 |  | 0.654 | 0.918 | 0.772 |
| Inner Mongolia | Western | 2.385 | 1.841 | 1.776 |  | 1.212 | 0.936 | 0.903 |
| Ningxia | Western | 0.351 | 0.218 | 0.221 |  | 2.522 | 1.566 | 1.589 |
| Qinghai | Western | 0.772 | 0.755 | 0.721 |  | 1.042 | 1.019 | 0.973 |
| Shandong | Eastern | 0.071 | 0.076 | 0.072 |  | 1.27 | 1.357 | 1.286 |
| Shanxi | Central | 4.793 | 4.242 | 4.775 |  | 1.076 | 0.952 | 1.072 |
| Shaanxi | Western | 2.784 | 1.374 | 1.656 |  | 1.827 | 0.902 | 1.087 |
| Shanghai | Eastern | 1.441 | 1.433 | 1.272 |  | 1.096 | 1.09 | 0.968 |
| Sichuan | Western | 8.163 | 14.423 | 22.883 |  | 0.302 | 0.534 | 0.848 |
| Tianjin | Eastern | 1.999 | 1.52 | 1.616 |  | 1.698 | 1.291 | 1.373 |
| Tibet | Western | 4.496 | 6.058 | 12.774 |  | 0.569 | 0.767 | 1.616 |
| Xinjiang | Western | 0.023 | 0.02 | 0.029 |  | 1.110 | 0.962 | 1.440 |
| Yunnan | Western | 0.095 | 0.128 | 0.086 |  | 0.892 | 1.201 | 0.809 |
| Zhejiang | Eastern | 0.647 | 0.869 | 0.639 |  | 0.790 | 1.061 | 0.780 |
| Chongqing | Western | 4.208 | 3.661 | 4.781 |  | 1.005 | 0.875 | 1.142 |
